# The Evaluation of the Efficacy and Safety of Midazolam Nasal Spray in Patients With Seizure Clusters: A Systematic Review and Meta-Analysis

**DOI:** 10.7759/cureus.34064

**Published:** 2023-01-22

**Authors:** Niraj Niraj, Sonia Mahajan, Ajay Prakash, Phulen Sarma, Bikash Medhi

**Affiliations:** 1 Pharmacology, Postgraduate Institute of Medical Education and Research, Chandigarh, IND; 2 Pharmacology, All India Institute of Medical Sciences, Jammu, IND

**Keywords:** systematic review, meta-analysis, midazolam nasal spray, seizure clusters, epilepsy

## Abstract

Midazolam nasal spray (MDZ-NS) is a new emerging rescue medication that suppresses epileptic seizures. Until now, few studies, pharmacokinetic (PK) and pharmacodynamic (PD) profiles, and clinical trials have shown that midazolam nasal spray could become an effective and promising alternative to conventional routes (intravenous {IV}/rectal). Therefore, we thought of conducting a systematic review and meta-analysis of midazolam (MDZ) to assess its potential outcomes. The analysis was also evaluated based on the pharmacokinetic (PK) and pharmacodynamic (PD) profiles of midazolam nasal spray.

A systematic literature search was carried out through various databases to identify studies of accounted outcomes of midazolam nasal spray (MDZ-NS). Randomized and other studies of patients (12 years or older) with seizure clusters (SCs) were included. A total of three full-text articles were considered for systematic review and meta-analysis as per the inclusion and exclusion criteria. The 5 mg MDZ-NS was observed to be equally safe as a placebo, and the risk ratio (RR) was 1.01 (95% confidence interval (CI): 0.67-1.53).

After the administration of MDZ-NS, either the patients remained seizure-free for six hours or more or the seizure was terminated within 10 minutes and had no recurrence between 10 minutes and six hours. The risk ratio (RR) obtained was 1.54 (95% CI: 1.25-1.91). The result was statistically significant as a higher success rate was observed with the use of 5 mg midazolam nasal spray compared to placebo (p < 0.0001). Heterogeneity was not observed in the results of the included studies (inconsistency index {I^2^}: 0%).

The present systematic review and meta-analysis demonstrated that 5 mg midazolam nasal spray was efficacious in treating patients with seizure clusters and is well-tolerated. Also, its use is relatively safe.

## Introduction and background

Epilepsy is a dysfunction of the brain characterized by an enduring predisposition to develop seizures [1]. The occurrence of a seizure is unpredictable and sometimes may increase the risk of trauma especially when it is associated with other neurological disorders such as Alzheimer’s and meningitis [2]; it may become life-threatening and further need hospitalization. According to the International League Against Epilepsy (ILAE) (2017), there are primarily three types of seizures: 1) generalized-onset seizures, 2) focal-onset seizures, and 3) unknown-onset seizures [3]. Approximately 50 million people have epilepsy worldwide; of these, around 80% of people live in low- and middle-income countries, such as India, which has 10 million cases. This figure reached around three million in the USA, while Europe has around six million [4]. It is predicted that 70% of cases of epilepsy can live seizure-free if they are timely diagnosed and treated appropriately.

When a seizure undergoes episodes or series of increased activity, broadly classified as seizure clusters (SCs). Seizure clusters are defined as “acute episodes of deterioration in seizure control.” They are also called acute repetitive seizures or flurries [5,6]. Some kind of epileptic seizure needs two or more anti-seizure medications [7], and because of taking multiple anti-seizure medications, sometimes, seizures do not successfully respond, and the consequence is having drug-resistant epilepsy or intractable epilepsy. Patients with intractable epilepsy are at high risk of having seizure clusters [5-7]. Almost one-third of seizures are drug-resistant [8]. And in case of no recovery in-between seizures, it may progress into status epilepticus, which becomes more critical and may lead to injury or death [8]. It has been reported that the prevalence of seizure clusters in outpatient and inpatient studies of patients having epilepsy is 13%-76% and 18%-61%, respectively [9,10]. It has been reported that patients having a history of seizure clusters have 2.5 times more risk for sudden unexplained death in epilepsy (SUDEP). However, seizure clusters alone may not be responsible for SUDEP risk, and it is likely only if there is a progression to the critical SUDEP threshold [11].

Several approved and well-known anti-seizure drugs such as sodium valproate, lamotrigine, levetiracetam, and benzodiazepine are available as therapeutic medications and are accepted worldwide, but the administration of these drugs is limited because in hospitals, it is typically administered by healthcare professionals. In emergency cases, the intravenous (IV) (lorazepam and diazepam), intramuscular (midazolam {MDZ}), or rectal (rectal diazepam) route is used, but intravenous and intramuscular routes are painful; also, many studies suggest that the rectal route is unacceptable due to social embarrassment. Oral (oral diazepam, clonazepam, or lorazepam) and buccal (buccal midazolam) routes are preferable outside and inside the hospital but may prolong the onset of action because of first-pass metabolism [12,13].

However, it is widely known that many seizure-related emergencies occur outside of the healthcare settings such as at home or in public places, so a convenient route of administration is essential, which has a rapid onset of action, is cost-effective, and is user-friendly to caregivers or non-health professionals. So, in such cases, the intranasal route is beneficial because it is painless, noninvasive, and easy to access [14,15]. But the intranasal route in nasal solution form is not standardized, and local irritation may occur because of the acidic solution (2.8-3.0 pH), and the small volume of the nasal cavity requires high doses, which leads to a significant first-pass effect and decreased bioavailability and efficacy [16-18]. So, the development of nasal spray formulation (midazolam nasal spray {MDZ-NS}) became a promising alternative in emergency cases as rescue medication where other routes are not feasible. It is also observed that the metabolic compound of midazolam nasal spray (MDZ-NS) to MDZ ratio for MDZ-NS is lower than oral MDZ, demonstrating that intranasal administration avoids first-pass metabolism [16-18].

FDA-approved in 2019, the first nasally administered spray (MDZ-NS) for treating seizure clusters is considered a rescue therapy in patients ≥12 years of age. It is proposed that MDZ-NS suppresses all types of epileptic seizures by increasing the gamma-aminobutyric acid (GABA) levels in the brain and can be absorbed consistently, rapidly, and extensively, which is crucial for seizure termination in seizure clusters to avoid the progression of status epilepticus [5,6,19].

In recent years, it has been shown from clinical trial studies that midazolam nasal spray could be an effective alternative to conventional routes (IV/rectal) in seizure cluster (SC) management. However, there is a paucity of such evidence-based studies. Therefore, we thought of conducting a systematic review and meta-analysis of midazolam nasal spray and published studies of the pharmacokinetic (PK) and pharmacodynamic (PD) profiles of the candidate to assess its efficacy and safety and understand more the potential benefits of this newer medication in clinical use in seizure cluster patients.

## Review

Materials and methods

A systematic review and meta-analysis were carried out as per the Preferred Reporting Items for Systematic Reviews and Meta-Analyses (PRISMA) guidelines [20]. The protocol was registered in the International Prospective Register of Systematic Reviews (PROSPERO) database (CRD42021275121). Randomized patients of seizure clusters (generalized and focal) using MDZ-NS were included in this meta-analysis. Confirmed cases of epilepsy experiencing seizure clusters and those with ages 12 years and above were included.

Data Collections

A systematic electronic search was carried out through PubMed/Medical Literature Analysis and Retrieval System Online (MEDLINE), Cochrane, Embase, Google Scholar, ScienceDirect, and Ovid databases to identify studies of accounted outcomes of midazolam nasal spray (MDZ-NS) for epileptic patients, which were available until 31 October 2022. All the full paper articles were carefully observed independently by all five authors. The search was carried out using keywords “midazolam,” “seizure,” “adult,” and “children” with the Boolean operator “AND” and using the Boolean operator NOT for “pregnant and children less than 12 years.” Moreover, for searching the data, post hoc filters have been applied. The Cochrane Collaboration tool (the Newcastle-Ottawa scale) was used to assess the risk of bias. The duplicate articles were excluded, and the remaining articles were included as per the eligibility criteria. The references to the included studies were also cross-checked, and there was no restriction on the publication date. In addition, gray literature was also performed in all possible studies after 31 October 2022. We assessed all the data including that of the National Informatics Centre (NIC) related to midazolam nasal spray (MDZ-NS) for seizure patients. Existing published data were reviewed by four authors (NN, SM, AP, and PS), and a hand search (manual scrutinization) was performed of included literature and relevant meta-analysis for reducing the risk of overlooking relevant literature. Any discrepancy was resolved by a unanimous approach with the fifth author (BM). Rejected studies were logically determined.

Selection of Study

The inclusion criteria were as follows: 1) confirmed and uncontrolled seizure patients, 2) randomized controlled trials (RCTs), 3) patients who were 12 years or older. The exclusion criteria were as follows: 1) observational studies, review studies, preclinical studies, and news reports; 2) literature other than English; and 3) unavailability of abstract or data.

Extraction of Data and Quality Assessment

The extraction of data was done independently by authors in pre-designed forms. The following data were extracted from the studies: first author, publication year, the number of patients and their mean age, country, study design, comorbidities, midazolam nasal spray (MDZ-NS) for the management of seizure, and concomitant medication received with corresponding mortality outcomes. Data from patients with confirmed seizures and controlled seizures were recorded separately.

Data Synthesis

All the patients were 12 years or older and had a limited number of RCTs, so the subgroup was not categorized as seizure patients for administering midazolam nasal spray (MDZ-NS). Meta-analysis was performed using the ReviewManager (RevMan) 5.1 software (Cochrane, London, England) for Windows. To account for the variability in studies, the random-effects model was used. Risk ratios (RR) were calculated for dichotomous data. Heterogeneity was evaluated by using the inconsistency index (I²) statistic. Relative risk was reported with a 95% confidence interval (CI), and a p-value of <0.05 was considered statistically significant. Forest plots were made for the outcomes assessed.

Types of Outcomes Measured

The primary outcomes were as follows: 1) potential benefits of the use of midazolam nasal sprays and 2) for a longer duration such as either the patients remained free of seizures for six hours or more or the seizure was terminated within 10 minutes and had no recurrence between 10 minutes and six hours after drug administration. The secondary outcomes were as follows: 1) adverse event profile due to the use of midazolam nasal spray and 2) long- and short-term safety profile.

Results

Study Selection

**Figure 1:**
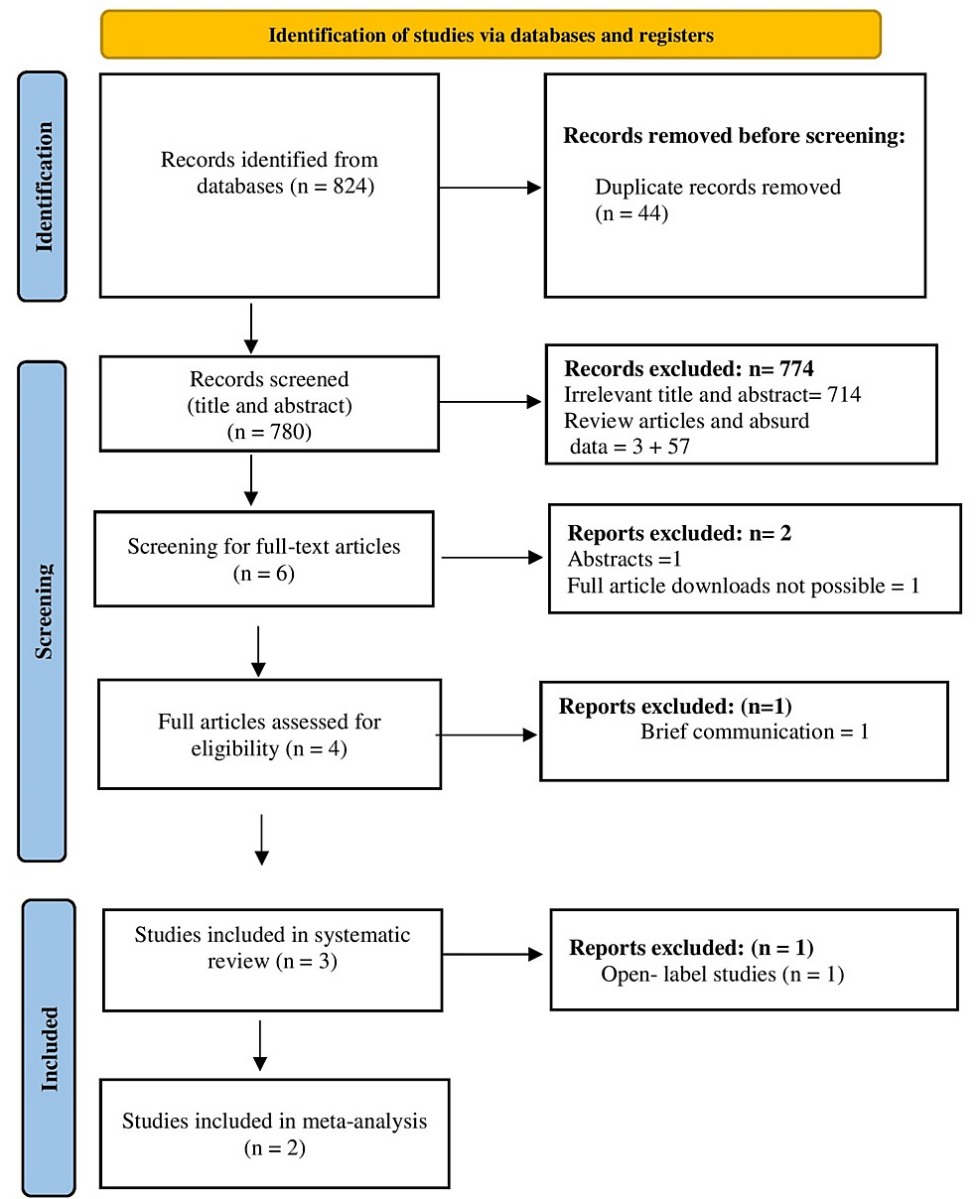
PRISMA flow chart PRISMA: Preferred Reporting Items for Systematic Reviews and Meta-Analyses

The study search results are depicted in Figure 1. After doing a database search, a total of 824 articles were obtained, from which 44 duplicate articles were found, which was not considered. Titles and abstracts of 780 articles were screened, from which 774 articles were excluded and further six full-text articles had been screened for eligibility. Of these, two articles were further excluded as one was a brief communication and the full text of the other article was not available in the public domain. Finally, three articles were included (as the study eligibility criteria) for systematic review and two for meta-analysis. The study design and characteristics of the patients in clinical trials are depicted in Table 1.

**Table 1:** Study design and patient characteristics included in the meta-analysis MDZ-NS, midazolam nasal spray; ITT, intention to treat; SD, standard deviation ^a^Patients may have been observed with >1 seizure type

Study	Clinical trial number	Study design	Treatment arms	Number of patients (ITT)	Age in years (mean ± SD)	Age groups, N (%)			Seizure type in the cluster, n (%)^a^
						≥12 years; <18 years	≥18 years; <65 years	≥65 years	Etiology (randomized safety population)
Detyniecki et al., 2019 [21]	NCT01390220	Phase III, double-blind, randomized, placebo-controlled trial; two phases (open-label test dose phase {TDP} and double-blind comparative phase)	5 mg MDZ-NS	134	34.0 ± 11.23	5 (3.7)	129 (96.3)	0	Focal impaired awareness: 71 (52.98)
									Focal to bilateral: 46 (34.32)
									Focal aware: 23 (17.16)
									Primary generalized tonic-clonic seizure: 8 (5.97)
									Tonic seizure: 8 (5.97)
									Absence seizure: 7 (5.22)
									Others: 7 (5.22)
									Myoclonic seizure: 3 (2.23)
									Atonic seizure: 2 (1.49)
			Placebo	67	31.5 ± 12.83	5 (7.5)	62 (92.5)	0	Focal impaired awareness: 33 (49.25)
									Focal to bilateral: 20 (29.85)
									Focal aware: 17 (25.37)
									Primary generalized tonic-clonic seizure: 7 (10.44)
									Tonic seizure: 3 (4.47)
									Absence seizure: 0 (0)
									Others: 0 (0)
									Myoclonic seizure: 3 (4.47)
									Atonic seizure: 0 (0)
Spencer et al., 2020 [22]	NCT01999777	Phase III, double-blind, randomized, placebo-controlled trial; three phases (screening phase ≤ 28 days, treatment phase ≤ 6 hours, and exit assessment ≤ 48 hours)	5 mg MDZ-NS	31	32.7 ± 12.9	2 (6.5)	29 (93.5)	0	Simple partial seizure: 13 (41.93)
									Complex partial seizure: 27 (87.09)
									Secondary generalized tonic-clonic seizure: 18 (58.06)
									Primary generalized tonic-clonic seizure: 3 (9.67)
									Absence seizure: 1 (3.22)
									Myoclonic seizure: 0 (0)
									Tonic seizure: 0 (0)
									Atonic seizure: 0 (0)
									Others: 1 (1.49)
			Placebo	31	35.9 ± 13.6	2 (6.5)	29 (93.5)	0	Simple partial seizure: 9 (29.03)
									Complex partial seizure: 26 (83.87)
									Secondarily generalized seizure: 17 (54.83)
									Primary generalized seizure: 4 (12.90)
									Absence seizure: 3 (9.67)
									Myoclonic seizure: 1 (3.22)
									Tonic seizure: 2 (6.45)
									Atonic seizure: 1 (3.22)
									Others: 1 (3.22)

Methodological Quality

**Figure 2:**
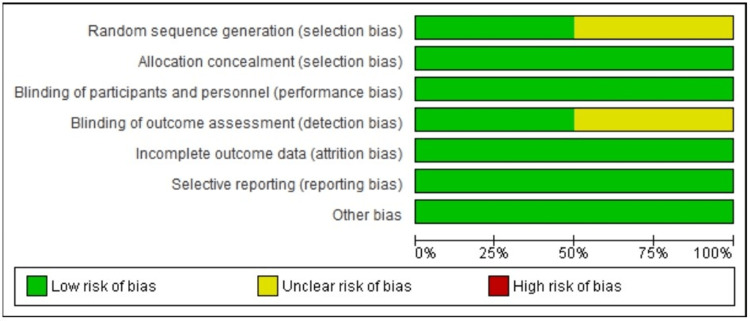
Risk of bias graph

The graph of the risk of bias assessment is shown in Figure 2. Some bias was observed in the randomization method used in one clinical trial. Also, there was some concern of bias in outcome assessment in one trial as information regarding blinding of the assessors was lacking.

Efficacy

**Figure 3:**
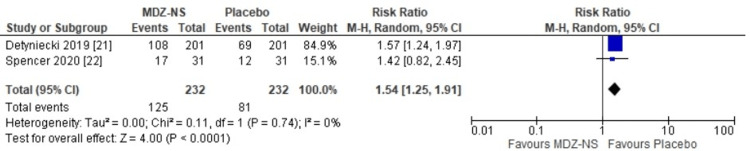
Risk ratios for treatment success with 5 mg midazolam nasal spray versus placebo MDZ-NS, midazolam nasal spray; CI, confidence interval; I^2^, inconsistency index; df, degrees of freedom

The primary outcome assessed in the present meta-analysis showed that there was a potential benefit of using 5 mg midazolam nasal spray (MDZ-NS); i.e., either the proportion of patients who responded remained seizure-free for six hours after treatment or seizure was terminated within 10 minutes, and no recurrence had been seen between 10 minutes and six hours after the treatment. The risk ratio (RR) obtained was 1.54 (95% CI: 1.25-1.91) (Figure 3). The overall result was statistically significant as a higher success rate had been observed with the use of 5 mg midazolam nasal spray (MDZ-NS) compared to placebo (p < 0.0001). No heterogeneity was detected in the results of the included studies (I^2^: 0%).

Also, in an open-label extension trial study by Wheless et al. (2019) (conducted between August 2012 and April 2017 in Australia, Canada, Germany, Hungary, Israel, Poland, Spain, Ukraine, and the USA; clinical trial registration number: NCT01529034; the median time spent by the patients being 16.8 months), 175 patients were enrolled for a total of 1998 SC episodes. Treatment success rates were met, and it was 55% (1108/1998) after a single dose of 5 mg MDZ-NS and 80.2% (617/769) after treatment of the second dose of 5 mg MDZ-NS. Treatment success was reproducible, and the overall treatment success was 86.3% (1725/1998) of treated episodes when one or two doses of 5 mg MDZ-NS were received by patients [23].

Safety

**Figure 4:**
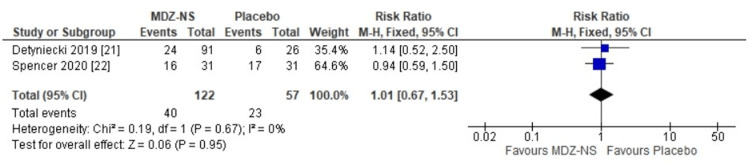
Risk ratios for treatment-emergent adverse events with 5 mg midazolam nasal spray versus placebo MDZ-NS, midazolam nasal spray; CI, confidence interval; I^2^, inconsistency index; df, degrees of freedom

**Figure 5:**
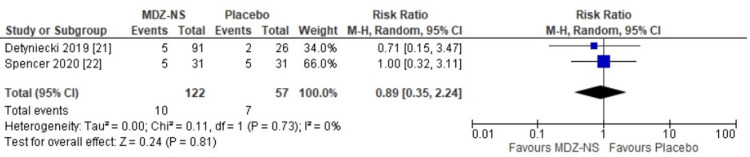
Risk ratios for nasal discomfort with 5 mg midazolam nasal spray versus placebo MDZ-NS, midazolam nasal spray; CI, confidence interval; I^2^, inconsistency index; df, degrees of freedom

**Figure 6:**
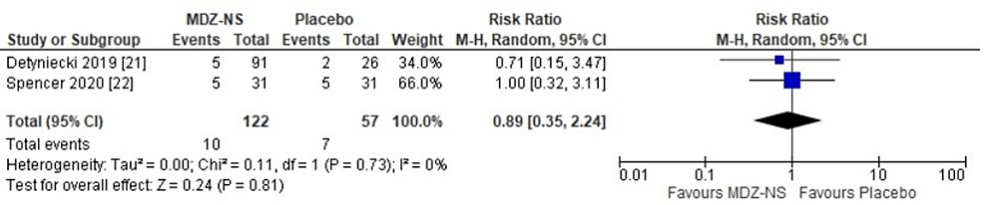
Risk ratios for somnolence with 5 mg midazolam nasal spray versus placebo MDZ-NS, midazolam nasal spray; CI, confidence interval; I^2^, inconsistency index; df, degrees of freedom

The 5 mg midazolam nasal spray was observed to be equally safe as a placebo (risk ratio: 1.01 {95% CI: 0.67-1.53}; p = 0.95) (Figure 4). No heterogeneity had been observed among the results of the included trials. The risk ratio for nasal discomfort observed with the use of midazolam nasal spray versus placebo was 0.89 (95% CI: 0.35-2-24), and the difference that had been observed was not statistically significant (p = 0.81) (Figure 5). A risk ratio of 3.17 (95% CI: 0.59-16.89) was obtained for somnolence with midazolam nasal spray versus that with the use of placebo; however, the finding was not relevant statistically (p = 0.18) (Figure 6). The adverse event profile is given in Table 2.

**Table 2:** Adverse event profile of midazolam nasal spray MDZ-NS, midazolam nasal spray; TEAE, treatment-emergent adverse event; SAE, serious adverse events

Study	Clinical trial number	Sample size	Drug	Control	Adverse effects	TEAE (%)	SAE (%)
Detyniecki et al., 2019 [21]	NCT01390220	292	5 mg MDZ-NS	Placebo-controlled	Nasal discomfort and somnolence were common, but most were mild intensity. Only two patients were considered clinically meaningful respiratory depression (one patient had this due to underlying diseases, and the other one had a history of sleep apnea)	51.4	4.8
Spencer et al., 2020 [22]	NCT01999777	62	5 mg MDZ-NS	Placebo-controlled	Nasal discomfort, nausea and throat irritation, and somnolence were the most common. All were mild cases. Out of the total MDZ-NS group, one patient (3.6%) reported suicidal intention, which was nonspecific	51.6	3.2
Wheless et al., 2019 [23]	NCT01529034	175	5 mg MDZ-NS		Nasal discomfort and somnolence were the most common but mild to moderate symptoms. Only four patients had serious TEAE convulsion, SAE, dysesthesia, or upper gastrointestinal hemorrhage (all were unlikely and not treatment-related effects)	57.1	11.2

Discussion

Randomized controlled trials comparing the midazolam nasal spray with a placebo (consisting of the same inactive ingredients of MDZ-NS) have been conducted, and only two RCTs (Detyniecki et al. [21] and Spencer et al. [22]) are available yet present. It is a new emerging drug for SCs and has been recently approved only in the USA as a rescue medication. To the best of the authors’ knowledge, the current study is the only systemic review and meta-analysis that is assessing the safety and efficacy of midazolam nasal spray in epileptic patients who are ≥12 years of age. Data are unavailable regarding safety and efficacy in children <12 years [21,22].

The results of this meta-analysis suggested that 5 mg midazolam nasal spray showed good efficacy over a placebo. As Spencer et al.’s study suggested, the patients get two seizures within six hours, and if left untreated, then approximately 65% of untreated patients get another seizure in the subsequent six hours [22]. Similar studies of other anti-epileptic drugs (AEDs) have been published where the seizure occurrence rate was 50%-76% in placebo-treated patients, but after midazolam nasal spray administration, there was a relative reduction of ≥35 percentage points [22]. Also, an open-label extension study of this present systemic review [23] revealed that the overall treatment success was 86.3% of treated episodes (1725/1998) after patients received single or double doses of 5 mg MDZ-NS.

In both, the RCTs’ treatment success consisted of two components: First is the seizure termination, and second, there was no seizure remission for up to six hours. Presently, we could not find the categorized percentage of effectiveness of MDZ-NS in different kinds of seizures, but studies by Vossler [24] and Brigo et al. [25] suggested that midazolam is effective in all kinds of seizures [24,25]. Also, the time to the first seizure following treatment is longer with midazolam nasal spray than with a placebo. More than 50% of patients respond to the first dose of midazolam and do not experience another seizure within 24 hours after the first [21,22].

The study shows that midazolam nasal spray (formulation) is relatively safe and well-tolerated. No serious adverse events have been reported among all candidate profiles. Nasal discomfort and somnolence conditions were commonly affected, but most of the time, they were of mild intensity (Table 2). Moreover, in the present study, the midazolam nasal spray was observed to be equally safe as a placebo in terms of the associated treatment-emergent adverse events (TEAEs). In it, a numerically lower risk of nasal discomfort was found with MDZ-NS compared to that of placebo. However, this was not statistically significant. Also, this study found the risk of somnolence to be numerically more with MDZ-NS than with a placebo.

Critical Evaluation Based on RCTs and Open-Label Studies

Indeed, the RCTs had been designed according to patient safety criteria: the patient who had participated had an established diagnosis of focal or generalized seizure or had a history of SCs and was only entitled to enrollment if their caregiver was able to take responsibility and trained to administer treatment, as well as recognize clusters. Each patient’s pattern was either evaluated and distinguished by an investigator as an individualized patient management plan (PMP) (members of the Epilepsy Study Consortium examined all PMP participants and approved trial participants as the protocol) or evaluated as seizure characterization in epilepsy monitoring unit (EMU) or preoperatively evaluated (such as admission had been planned within 28 days). Upon EMU admission, patients entered pre-treatment observation and were continuously observed until treatment protocols were met [21,22].

However, the authors revealed from Detyniecki et al.’s study that efficacy outcomes were decided primarily from the caregiver’s response such as seizure-related information, trial drug administration, or the safety data record, but it is important to note that caregivers were a non-healthcare professional and they were trained for the trial purpose only, and the trained staff had been contacted only in case of emergency. Also, a double-blind trial was followed by open-label 5 mg MDZ-NS when the cluster did not terminate seizure within 10 minutes or another seizure occurred up to six hours after the initial drug administration. Also, an open-label extension trial study (Wheless et al. [23]) proved the meaningful effect of 5 mg MDZ-NS, but the major drawback of the study was that there was a lack of a control group. Moreover, Spencer et al.’s study also observed that the treatment difference between MDZ-NS and placebo was significant and clinically meaningful; this study also had certain flaws such as the population enrolled in the EMU setting differed from the population for which MDZ-NS was approved. Even increased seizure activity in an EMU setting may be different from naturally occurring seizure clusters (such as withdrawal of AEDs and sleep insomnia), Also, the EMU setting has provided a unique environment that may differ from the natural environment (regarding the safety of the patient) and was continuously monitored by professional healthcare staff.

Analysis Based on PK and PD Profile of Midazolam Nasal Spray

The 5 mg midazolam nasal spray (MDZ-NS) single-dose formulation has been developed as a ready-to-use treatment option in inpatient and outpatient settings. It delivers the therapeutic dose in a small volume (0.1 mL) in the pH range of 5.0-9.0. [23], so the untoward effect of nasal irritation is minimum. A few hundred microliters of the drug are delivered intranasally due to the limited drug (150-250 µL)-holding capacity of each nostril [26]. Therefore, an atomizer is required to achieve effective drug absorption [13]. Because of the desired potential effect and limited dose of drug delivery in the nostril, 5 mg midazolam nasal spray is recommended. The midazolam nasal spray is given as a single spray (5 mg per dose) in one nostril, which is clinically meaningful in patients having epilepsy. If patients did not respond to the first 5 mg of MDZ-NS, then it was given a second dose of 5 mg in the opposite nostril after a gap of 10 minutes, but not more than 10 mg should be advised to treat a single episode.

Pharmacokinetic (PK) and pharmacodynamic (PD) data suggested that up to 20 mg of MDZ-NS is safe and well-tolerated in humans [18,26,27]. The maximum concentration (Cmax) and area under the curve (AUC) have been increased proportionally with the dose of MDZ-NS up to 15 mg, but after that, its relative bioavailability is reduced, and almost constant in plasma exposure has been observed.

In UCB Pharma data, pop PK model [18], the PK of MDZ-NS in the phase I trial study was conducted in healthy participants and patients (adult and pediatric) with epilepsy, and the peak plasma drug maximum concentration (Cmax) and area under the plasma concentration-time curve (AUC0-∞) increased proportionally with dose up to 5 mg and were higher in healthy older patients (≥65 years) than younger patients (18-40 years), but the time to reach peak plasma concentration (Tmax) was almost similar in both age groups.

The study also demonstrated that the time to maximum concentration (Tmax) in adult healthy individuals irrespective of their age is 14.5-17.3 minutes up to 5 mg and, in an adult with epilepsy, it was 9-21.5 minutes up to 10-20 mg of MDZ-NS, whereas elimination half-life (T1/2) was 3.7-4.7 hour shorter than the healthy individuals (6-8 hours) [18,28]; it could be enzyme-inducing AEDs in patients with epilepsy.

The PK up to 5 mg MDZ-NS in pediatric patients (2-13 years) with epilepsy was assigned by body weight. The Tmax was 15 minutes for all cohorts, and AUC0-∞ was 75.2 ng.hour/mL, but it was lower in <5 mg of MDZ-NS [18]. In an analysis of UCB Pharma (2019), The Cmax and AUC0-∞ in 5 mg MDZ-NS to healthy individuals were found to be 54.7 ± 30.4 ng/mL and 126.2 ± 59 ng.hour/mL, respectively, and the median Tmax was 17.3 minutes (7.8-28.2 minutes) [26].

MDZ-NS reaches in the cerebral cortex within 2-5 minutes, which is significantly important in SC cases. The mean absolute bioavailability of 5 mg MDZ-NS is 44%. In adult and pediatric patients, it can be bound with 97% plasma protein and has a volume of distribution of 226.5 L. And MDZ-NS has a median elimination half-life ranging from 2.1 to 6.2 hours (it varies with the age but is independent of dose) [27,28].

Pharmacokinetic effects due to intrinsic factors (age, sex, race, and body weight) and extrinsic factors (such as CYP3A inducers) are not clinically significant factors for MDZ-NS dosing ≤20 mg, but a US products label suggested avoiding coadministration with moderate or strong CYP3A4 inhibitors [27].

In adults, healthy individuals, and patients with epilepsy irrespective of their age, PD effects (sedation and psychomotor impairment) of 5 mg MDZ-NS are not significantly different and suggest that shorter T1/2 may not be clinically relevant. The peak effect on sedation of 5 mg MDZ-NS was within 15-20 minutes, after a single- or double-dose administration, and only returned to near baseline levels after 4 hours, whereas the peak effect on psychomotor impairment in healthy adults and adults with epilepsy was 17 minutes to two hours post-dose and returns to the baseline within 240 minutes. It was significantly greater in 5 mg than in 2.5 mg, but no significant difference was observed between 15 and 20 mg MDZ-NS [18,28]. Midazolam is regulated under Schedule 4 of the Controlled Substances Act. It may consider abuse potential because of the benzodiazepine class. Given the short-term use of the midazolam nasal spray, the abuse liability is relatively very low, and the development of tolerance was not observed [18]. As per PK and PD data analysis, midazolam nasal spray (MDZ-NS) is not suggested to be used as regular medicine for a longer duration, so the probability of similar adverse events such as the risk of suicidal thoughts and the development of the deterioration of depressive symptoms as in the other classes of benzodiazepine drugs is rare, and there is no evidence for them to occur [18,26].

Overall, in this current study, the observed treatment seems meaningful and clinically significant and will make it easier for seizure patients and potentially accessible in case of emergency. However, the total sample of the study is very small; if more data will be available in the future, it can be analyzed more coherently, but the analysis of PK and PD and previous studies (Scheepers et al. [17], Wheless [18], and Bouw et al. [27]) emphasizes the effectiveness of the treatment agent. However, midazolam nasal spray has some contraindications (such as acute narrow-angle glaucoma, hypersensitivity to midazolam, and the patient who is taking opioid medication), so its use should be administered cautiously. There may be discomfort in using it in patients who are suffering from conditions such as obstruction in nasal passages such as nasal polyps, septal defects, allergic rhinitis, or nasal trauma [29,30]. So, the physicians who prescribed the midazolam nasal spray should look if the patients have any contraindication or contraindication not related to midazolam.

Limitations

Although the randomized, double-blind, placebo-controlled trial of Detyniecki et al.’s study is considered more important because it contributed to a greater number of patients (N = 292) than the other RCT (Spencer et al.’s study, N = 62), the overall sample size is small.

RCTs comparing MDZ-NS with other standard anti-seizure drugs were not found as adequately powered, and blinded comparative trials between them would be complicated; it could be due to different routes of administration. Recently, the meta-analysis by Chhabra et al. (2021) [30] is published, which compared intranasal midazolam nasal solution to intravenous/rectal benzodiazepines (where intranasal midazolam was used as nasal drop formulation). The authors also intended to perform subgroup analysis depending on the dose and the duration of the studies. However, it could not be performed due to a lack of data.

## Conclusions

The result of the present systematic review and meta-analysis and the analysis based on the evidence of PK and PD profiles of MDZ-NS (such as rapid Tmax, consistent absorption, rapid distribution to the CNS, peak sedative effect, psychomotor impairment, return to baseline function, the lack of first-pass metabolism, and minimum potential drug-drug interaction) demonstrated that it is safe, well-accepted, and well-tolerated, and no potentially serious adverse effects have been reported. Therefore, it is expected that midazolam nasal spray is an effective alternative to currently established drugs as a rescue medication for seizure clusters, which may fulfill the unmet need of the current clinical epileptic regimen.

## References

[1] Scheffer IE, Berkovic S, Capovilla G (2017). ILAE classification of the epilepsies: position paper of the ILAE Commission for Classification and Terminology. Epilepsia.

[2] Kerr MP (2012). The impact of epilepsy on patients' lives. Acta Neurol Scand Suppl.

[3] Fisher RS, Cross JH, D'Souza C (2017). Instruction manual for the ILAE 2017 operational classification of seizure types. Epilepsia.

[4] Sen A, Capelli V, Husain M (2018). Cognition and dementia in older patients with epilepsy. Brain.

[5] Haut SR (2015). Seizure clusters: characteristics and treatment. Curr Opin Neurol.

[6] Jafarpour S, Hirsch LJ, Gaínza-Lein M, Kellinghaus C, Detyniecki K (2019). Seizure cluster: definition, prevalence, consequences, and management. Seizure.

[7] Sankaraneni R, Lachhwani D (2015). Antiepileptic drugs--a review. Pediatr Ann.

[8] Berg AT, Vickrey BG, Testa FM, Levy SR, Shinnar S, DiMario F, Smith S (2006). How long does it take for epilepsy to become intractable? A prospective investigation. Ann Neurol.

[9] Haut SR, Nabbout R (2022). Recognizing seizure clusters in the community: the path to uniformity and individualization in nomenclature and definition. Epilepsia.

[10] Chen B, Choi H, Hirsch LJ (2017). Prevalence and risk factors of seizure clusters in adult patients with epilepsy. Epilepsy Res.

[11] Ochoa-Urrea M, Lacuey N, Vilella L (2021). Seizure clusters, seizure severity markers, and SUDEP risk. Front Neurol.

[12] Kälviäinen R (2015). Intranasal therapies for acute seizures. Epilepsy Behav.

[13] Mula M (2017). New non-intravenous routes for benzodiazepines in epilepsy: a clinician perspective. CNS Drugs.

[14] Arora P, Sharma S, Garg S (2002). Permeability issues in nasal drug delivery. Drug Discov Today.

[15] Ugwoke MI, Verbeke N, Kinget R (2001). The biopharmaceutical aspects of nasal mucoadhesive drug delivery. J Pharm Pharmacol.

[16] Kapoor M, Cloyd JC, Siegel RA (2016). A review of intranasal formulations for the treatment of seizure emergencies. J Control Release.

[17] Scheepers M, Scheepers B, Clarke M, Comish S, Ibitoye M (2000). Is intranasal midazolam an effective rescue medication in adolescents and adults with severe epilepsy?. Seizure.

[18] Wheless JW (2021). A critical evaluation of midazolam nasal spray for the treatment of patients with seizure clusters. Expert Rev Neurother.

[19] Mellion SA, Bourne D, Brou L, Brent A, Adelgais K, Galinkin J, Wathen J (2017). Evaluating clinical effectiveness and pharmacokinetic profile of atomized intranasal midazolam in children undergoing laceration repair. J Emerg Med.

[20] Moher D, Liberati A, Tetzlaff J, Altman DG (2010). Preferred reporting items for systematic reviews and meta-analyses: the PRISMA statement. Int J Surg.

[21] Detyniecki K, Van Ess PJ, Sequeira DJ, Wheless JW, Meng TC, Pullman WE (2019). Safety and efficacy of midazolam nasal spray in the outpatient treatment of patients with seizure clusters-a randomized, double-blind, placebo-controlled trial. Epilepsia.

[22] Spencer DC, Sinha SR, Choi EJ (2020). Safety and efficacy of midazolam nasal spray for the treatment of intermittent bouts of increased seizure activity in the epilepsy monitoring unit: a double-blind, randomized, placebo-controlled trial. Epilepsia.

[23] Wheless JW, Meng TC, Van Ess PJ, Detyniecki K, Sequeira DJ, Pullman WE (2019). Safety and efficacy of midazolam nasal spray in the outpatient treatment of patients with seizure clusters: an open-label extension trial. Epilepsia.

[24] Vossler DG (2020). The nose knows: intranasal midazolam to treat acute seizures during inpatient epilepsy monitoring. Epilepsy Curr.

[25] Brigo F, Nardone R, Tezzon F, Trinka E (2015). Nonintravenous midazolam versus intravenous or rectal diazepam for the treatment of early status epilepticus: a systematic review with meta-analysis. Epilepsy Behav.

[26] (2022). UCB, Inc. NAYZILAM® (midazolam) nasal spray, CIV US prescribing information. https://www.ucb-usa.com/_up/ucb_usa_com_kopie/documents/Nayzilam_PI.pdf.

[27] Bouw MR, Chung SS, Gidal B, King A, Tomasovic J, Wheless JW, Van Ess PJ (2021). Clinical pharmacokinetic and pharmacodynamic profile of midazolam nasal spray. Epilepsy Res.

[28] Berg AK, Myrvik MJ, Van Ess PJ (2017). Pharmacokinetics, pharmacodynamics, and tolerability of USL261, midazolam nasal spray: randomized study in healthy geriatric and non-geriatric adults. Epilepsy Behav.

[29] Burstein AH, Modica R, Hatton M, Forrest A, Gengo FM (1997). Pharmacokinetics and pharmacodynamics of midazolam after intranasal administration. J Clin Pharmacol.

[30] Chhabra R, Gupta R, Gupta LK (2021). Intranasal midazolam versus intravenous/rectal benzodiazepines for acute seizure control in children: a systematic review and meta-analysis. Epilepsy Behav.

